# Biosecurity Implications, Transmission Routes and Modes of Economically Important Diseases in Domestic Fowl and Turkey

**DOI:** 10.3390/vetsci12040391

**Published:** 2025-04-21

**Authors:** László Kovács, Gerda Domaföldi, Pia-Charlotte Bertram, Máté Farkas, László Péter Könyves

**Affiliations:** 1 Department of Animal Hygiene, Herd Health and Mobile Clinic, University of Veterinary Medicine, H1078 Budapest, Hungary; pia-charlotte.bertram@student.univet.hu (P.-C.B.); konyves.laszlo@univet.hu (L.P.K.); 2 Poultry-Care Kft., H5052 Újszász, Hungary; farkas.mate@univet.hu; 3 National Laboratory of Infectious Animal Diseases, Antimicrobial Resistance, Veterinary Public Health and Food Chain Safety, University of Veterinary Medicine, H1078 Budapest, Hungary; 4 Department of Digital Food Science, Institute of Food Chain Science, University of Veterinary Medicine, H1078 Budapest, Hungary

**Keywords:** poultry diseases, transmission routes, biosecurity, public health, poultry industry, food safety, zoonosis

## Abstract

The poultry industry is a critical source of affordable protein worldwide; however, it faces continuous threats from various poultry diseases that significantly impact public health, economic stability, and food security. Knowledge of and examination of the transmission routes, risk factors, and environmental survival characteristics of the most important pathogens affecting poultry populations, as well as the importance of strict biosecurity, are pivotal. Transmission routes are split into direct and vector-borne pathways, and indirect ways, which include infections via contaminated surfaces and vector-borne pathways, including insects and rodents. Avian influenza virus and Newcastle disease virus spread through respiratory droplets, and their transmission risk increases with increasing stocking density. While other pathogens (e.g., infectious bursal disease virus and *Salmonella* spp.), to persist long-term in the environments, for example, feed and litter, increasing the probability to persist long-term in the environments, for example, feed and litter, increasing the probability of infection. The long-term resilience of pathogens in multiple pathogens in various environmental conditions highlights the role of biosecurity, sanitation, and hygiene controls in preventing disease outbreaks. High stocking density in production systems, suboptimal ventilation, and inadequate biosecurity controls further increase transmission risks. This paper summarizes important disease transmissions and reinforces the need for strict biosecurity protocols and routine health monitoring to prevent the spread of pathogens within and beyond poultry facilities. These strategies can support safe poultry production, address growing global demand, and ensure food safety and public health.

## Simple Summary

Poultry is one of the most important sources of affordable protein worldwide; however, the industry faces serious challenges from diseases that can harm both animal and human health, disrupt food supplies, and cause economic losses. This study focuses on how these diseases spread among birds through different channels. Some viruses, like avian influenza and Newcastle disease, spread through the air, especially in crowded conditions. Others, like Salmonella, can survive for long periods in feed, litter, and the environment, increasing the risk of future outbreaks. The study shows that poor ventilation, overcrowding, and weak hygiene practices exacerbate the problem. To address this, the authors highlight the need for strong disease prevention measures, such as regular cleaning, health checks, and strict rules to prevent pathogens from entering or spreading within poultry farms. These actions are essential to protect public health, make poultry farming more secure, and meet the growing global demand for safe and healthy food.

## 1. Introduction

The poultry industry provides a significant and vital protein affordable protein source, offering meat and eggs to meet global nutritional needs [1,2]. Poultry is a cost-effective source of protein due to its low production cost, short production cycle, and widespread global availability [3,4]. Additionally, to the financial factor, poultry meat and eggs are highly nutritious, providing a healthy source of protein for muscle development and a vital supply of B-complex vitamins, which play crucial roles in energy metabolism and brain function [5,6,7]. These qualities make them particularly attractive to health-conscious consumers [8]. The demand for poultry products is expected to increase steadily [4]. The Organization for Economic Co-operation and Development (OECD) and the Food and Agriculture Organization of the United Nations (FAO) stated together in 2024 that by 2033, global poultry consumption is expected to reach 160 million tons (ready-to-cook), making up half of the increase in total meat consumption. Poultry meat alone provides 43% of all protein consumed from meat sources [9]. Nevertheless, the increase in production is associated with increased risks to public health, economic losses, and food security [10]. Disease outbreaks can cause significant economic losses and threaten food security by leading to shortages of poultry products and causing inflation. The cost and time associated with the control and minimization of the spread of outbreaks, including the culling of infected flocks, can be substantial. These challenges threaten the food supply of both local and global markets [11].

As consumer awareness of dietary intake continues to increase, the significance of maintaining animal health and ensuring appropriate husbandry practices has become progressively more prominent. In this context, the implementation of robust biosecurity measures is pivotal, serving as a critical safeguard for the ethical and hygienic production of animal-derived food products [12,13,14].

Biosecurity practices are a fundamental component of sustainable livestock production, promoting animal health and welfare and thereby reducing reliance on pharmacological interventions, particularly antibiotics. This is of increasing importance in light of global concerns regarding antimicrobial resistance [15] and its associated risks to public health [16]. By preventing pathogen introduction and limiting disease transmission within animal populations, biosecurity measures contribute significantly to the production of meat with minimal additive use, aligning with both food safety and public health objectives.

Biosecurity includes a series of preventive measures and protocols designed to minimize the risk of infection, pathogen introduction, and spread of pathogens within farms, humans, and wildlife [17]. The key focus is controlling access to poultry facilities and maintaining strict hygiene protocols; health monitoring of the flock by a veterinarian is necessary, and waste management systems and proper feed storage help reduce contamination risks and decrease vector-borne transmissions [18]. Following biosecurity protocols and rules helps minimize economic losses caused by disease outbreak management, protects public health [19,20], and contributes to the long-term sustainability and resilience of poultry production systems [21].

Proper pest control reduces the damage caused by pests and limits the spread of diseases carried and spread by flies, birds, or rodents. This leads to improved livestock health and farm hygiene [19,22].

**Figure 1:**
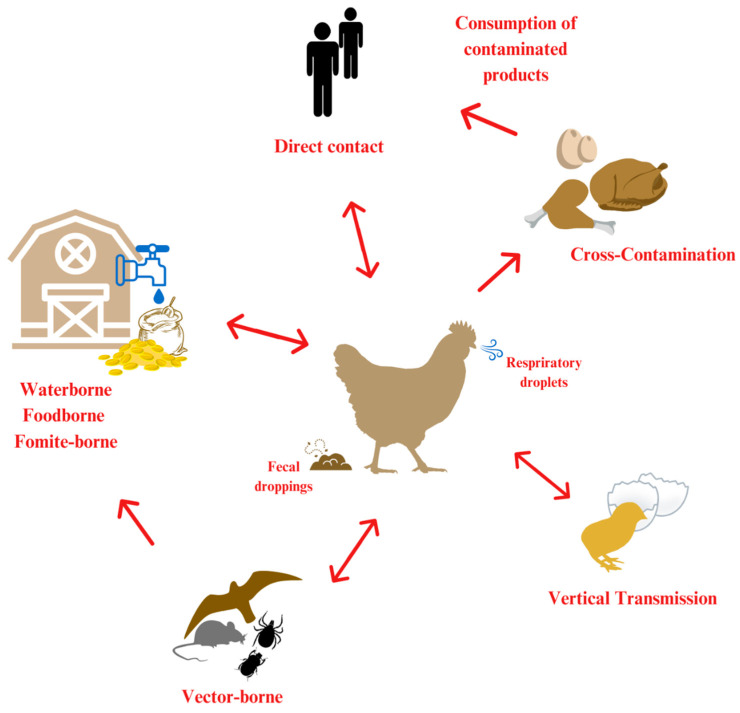
The different transmission pathways of zoonotic poultry pathogens include horizontal and vertical transmission, direct and indirect transmission, and cross-contamination [32]. The arrow pointing in one direction is meant to indicate one-way transmission, while the arrow pointing in both directions is meant to indicate transmission in both directions.

Understanding the transmission of pathogens is important to minimize their spread and infection, which is crucial for effective control and prevention [23]. Figure 1 illustrates the possible transmission routes of poultry pathogens. Direct transmission occurs when pathogens spread through direct contact between infected and healthy birds [19]. For instance, pathogens of avian influenza (AI) and Newcastle disease (ND) are often spread by direct contact, which usually occurs in intensive farming environments due to the high density of birds [24,25,26]. The spread of pathogens via contaminated surfaces, equipment, food, water, and fomite transmission is indirect [19,27]. This transmission route is incredibly challenging because pathogens can often survive for an extended period under multiple environmental conditions, facilitating widespread outbreaks [28]. Human movement, ventilation, and communal water systems are critical factors in the spread of disease [26,29]. Additionally, vector-borne transmission is of great importance, as wildlife, rodents, and insects can carry pathogens. Therefore, strict biosecurity is of great importance [30,31].

The main objective of this review is to provide a comprehensive overview of the major transmission routes of poultry pathogens and highlight their significance in relation to disease prevention and biosecurity. By examining each pathway, this review aims to support the development and implementation of effective biosecurity strategies that can be applied to breeding and commercial poultry operations.

## 2. Pathogens

The health and productivity of poultry are constantly threatened by various pathogens that can lead to economic losses by increasing mortality and morbidity [33]. These pathogens are categorized as viral, bacterial, and parasitic and pose a significant challenge to the poultry flock health and farm profitability worldwide [34,35,36].

### 2.1. Viral Pathogens

The AI, ND, and Marek’s disease (MD) viruses are considered to be three of the most lethal pathogens affecting poultry populations [37]. Among these, AI is a zoonotic disease that poses a high risk to both domestic poultry and wild bird populations, with the potential to impact human health [38]. The clinical presentation of AI varies depending on the strain. Highly pathogenic avian influenza (HPAI) causes severe symptoms, including respiratory signs (such as coughing, sneezing, and nasal discharge), as well as neurological and digestive disorders, often resulting in high mortality rates in infected birds. In contrast, low pathogenic avian influenza (LPAI) may be asymptomatic or cause only mild respiratory symptoms [39]. AI can be transmitted through multiple routes and is capable of surviving in cold environments, making it a highly transmissible virus that threatens public health, food safety, and economic stability [40,41].

Similarly, Newcastle disease virus (NDV) is a highly contagious pathogen that affects various poultry species worldwide and causes substantial economic losses [42]. Similarly, NDV is a highly contagious pathogen that affects various poultry species worldwide and causes substantial economic losses [43]. Some strains of NDV may cause asymptomatic infections, increasing the risk of undetected transmission [44].

Infectious bronchitis virus (IBV) is another significant challenge that causes highly contagious respiratory diseases. Depending on the strain, IBV can also affect the gastrointestinal, urinary, and reproductive systems, resulting in decreased egg production and economic losses [45]. Its high transmission rate is influenced by poultry population density, environmental conditions, and the level of biosecurity [46].

Infectious bursal disease (IBD) primarily targets the immune system, rendering birds vulnerable to secondary infections. The virus is highly resistant to disinfectants and can survive over a wide pH range (2–12) and at elevated temperatures. These characteristics contribute to its environmental persistence and high transmissibility [47].

The infectious laryngotracheitis (ILT) virus mainly affects the respiratory system of chickens, causing severe respiratory distress, decreased productivity, and, in severe cases, high mortality [48].

Marek’s disease virus (MDV) is another major concern. It is highly contagious and causes immunosuppression and tumor formation in poultry [49]. Vaccination reduces clinical signs but does not prevent viral transmission, allowing the virus to continue spreading in vaccinated flocks [47].

Avian metapneumovirus (aMPV), formerly known as avian pneumovirus (APV) or avian rhinotracheitis (ART) virus, is a highly contagious RNA virus that affects turkeys, chickens, and ducks [50]. It causes respiratory and reproductive tract infections, leading to decreased egg production, poor weight gain, and increased vulnerability to secondary infections [51]. aMPV is the causative agent of swollen head syndrome (SHS) in chickens and turkey rhinotracheitis (TRT) in turkeys. SHS may present with mild respiratory and reproductive symptoms or, in severe cases, neurological signs [52].

Fowlpox, caused by the fowlpox virus (FWPV), is a globally distributed disease of chickens and turkeys. It manifests in two forms: the cutaneous form, characterized by lesions on unfeathered skin, and the more severe diphtheritic form, which affects the respiratory and gastrointestinal tracts and can lead to asphyxiation [47].

Avian encephalomyelitis virus (AEV) is a highly stable picornavirus that primarily affects the central nervous system and other internal organs of poultry. It spreads via both horizontal (mainly fecal-oral) and vertical transmission [53]. Young birds exhibit neurological signs, such as depression, tremors, and ataxia, while adult chickens may be asymptomatic or experience reduced egg production and hatchability [54].

Chicken infectious anemia (CIA), caused by the chicken anemia virus (CAV), is a globally important immunosuppressive disease that results in substantial economic losses [55]. Clinical signs include pale combs and wattles due to anemia (packed cell volume < 27%), anorexia, lethargy, skin hemorrhages, and poor weight gain. These effects are often exacerbated by secondary bacterial infections [47].

Avian reoviruses (ARVs) are widespread in poultry. While many strains are non-pathogenic and commonly found in healthy birds, some cause disease, with viral arthritis or tenosynovitis being the most significant, particularly in broiler chickens [56].

Fowl adenovirus (FAdV) is a prevalent viral pathogen that causes severe diseases in chickens, including inclusion body hepatitis, hepatitis hydropericardium syndrome, gizzard erosion, and ulceration [57]. Infected birds may exhibit anorexia, depression, ruffled feathers, huddling, and greenish diarrhea. FAdV spreads via both aerosol and vertical transmission [58,59,60].

Egg drop syndrome 76 (EDS 76) is a viral disease caused by an avian adenovirus that results in the production of thin-shelled, soft-shelled, or shell-less eggs in otherwise healthy birds [61]. It spreads through both horizontal and vertical transmissions, leading to major production losses in commercial laying flocks. EDS 76 occurs in three forms: classic, endemic, and sporadic forms. Classic EDS 76 results from vertical transmission from the infected breeding stock. Endemic EDS 76 spreads horizontally via contaminated egg trays, crates, vehicles, and shared egg packing equipment. Sporadic cases often originate from contact with ducks, geese, or water sources contaminated by wild birds, which can lead to persistent endemic infections [47].

### 2.2. Bacterial Pathogens

Bacterial pathogens play a critical role in poultry health and production, with avian pathogenic *Escherichia coli* (APEC) being one of the most prevalent causes of bacterial infections [62]. Understanding the rapid spread of these pathogens is essential due to their significant consequences for both animal and public health, often leading to increased use of antimicrobial drugs [63]. *E. coli* is an opportunistic pathogen that normally exists as part of the gut microflora and is generally harmless. However, under stress or immune suppression, it can become pathogenic and cause colibacillosis. This disease can manifest in various forms, including airsacculitis, cellulitis, peritonitis, salpingitis, swollen head syndrome, omphalitis, and pericarditis [47].

Similarly, *Mycoplasma gallisepticum* causes considerable economic losses worldwide. It negatively affects weight gain, feed conversion efficiency, egg production, hatchability, and embryo viability, and increases condemnation rates at slaughter [64,65].

Pullorum disease and fowl typhoid are two major poultry diseases caused by *Salmonella pullorum* and *Salmonella gallinarum*, respectively. These pathogens primarily affect chickens and turkeys. In chicks and poults, the clinical signs include anorexia, diarrhea, dehydration, weakness, and high mortality. Infections in mature birds lead to reduced egg production, fertility, and hatchability, as well as anorexia and elevated mortality. Gross and microscopic lesions include hepatitis, splenitis, typhlitis, omphalitis, myocarditis, ventriculitis, pneumonia, synovitis, peritonitis, and ophthalmitis [66,67]. Salmonella species are particularly important due to their zoonotic potential and the significant role they play in foodborne illnesses globally [32,68]. *Salmonella Enteritidis* and *Salmonella Typhimurium* are major zoonotic pathogens in poultry production, often causing human infections through the consumption of contaminated poultry products [69,70]. Other notable Salmonella species include *Salmonella enterica* and *Salmonella Infantis*. The latter is increasingly prevalent in broiler flocks and frequently linked to human salmonellosis cases [71].

Fowl cholera, caused by *Pasteurella multocida*, is a highly contagious disease in chickens. Its severity and prevalence depend on factors such as the species and age of the host, environmental conditions, and virulence of the bacterial strain [72].

*Campylobacter jejuni* causes campylobacteriosis in poultry and is a leading cause of foodborne illness in humans. Transmission often results from cross-contamination during processing, improper handling, or undercooking of poultry meat, making it a major concern for food safety [73].

*Staphylococcus aureus* is another significant opportunistic pathogen. While typically harmless and part of the normal skin and mucosal microflora in poultry, under certain conditions, such as stress or immunosuppression, it can cause infections like bumblefoot, omphalitis, and arthritis [74]. Tolba et al. (2008) [75] reported contamination in 81.75% of surveyed poultry farms. Excessive antibiotic use has contributed to the emergence of methicillin-resistant *Staphylococcus aureus* (MRSA) strains in poultry. These strains pose serious food safety and public health risks as they can be transmitted to humans through direct contact, environmental exposure on farms, or consumption of contaminated poultry products [75,76,77].

*Erysipelothrix rhusiopathiae* is an important zoonotic pathogen with considerable economic impact. Although outbreaks are sporadic, they can cause high mortality rates, decreased productivity, and significant control and biosecurity costs [78,79].

Necrotic enteritis is a clostridial disease caused by *Clostridium perfringens*, particularly in broiler chickens. Although it is a part of the normal gut flora, imbalances in the intestinal environment or bacterial overgrowth can trigger toxicosis [80,81]. This leads to toxin production, reduced growth performance, and poor feed conversion. Necrotic enteritis frequently occurs following *Eimeria* spp. infections, which predispose the gut to bacterial overgrowth [82,83].

*Mycoplasma synoviae* (*MS*) is another important bacterial pathogen responsible for respiratory infections, synovitis, and reproductive disorders in poultry. MS infections result in economic losses due to stunted growth, elevated mortality, reduced egg production, and increased carcass condemnation rates in slaughterhouses [84,85].

### 2.3. Parasitic Pathogens

Parasitic pathogens also pose significant challenges to poultry health management. *Ornithonyssus sylviarum*, commonly known as the northern fowl mite, is a frequent external parasite in poultry flocks [86]. Infestations can lead to blood loss-induced anemia, reduced egg production, and a general decline in flock health and welfare [87].

*Dermanyssus gallinae*, known as the poultry red mite, is another major ectoparasite in the poultry industry. It adversely affects animal health, welfare, and production efficiency. These mites feed on the blood of birds, causing substantial blood loss, anemia, decreased egg production, and increased stress. In addition to their direct harm, they also act as vectors for bacterial and viral pathogens, exacerbating the difficulty of managing disease outbreaks on poultry farms [88].

*Histomonas meleagridis* is the causative agent of histomoniasis, also referred to as blackhead disease. It is particularly severe in turkeys, where the infection causes inflammation and ulceration of the cecal wall, which may lead to peritonitis, liver inflammation, and tissue necrosis [89,90].

Coccidiosis, caused by *Eimeria* spp., is one of the most prevalent parasitic diseases in intensive poultry farming systems. It often results in hemorrhagic diarrhea, weight loss, and poor feed conversion efficiency. These parasites flourish in contaminated environments, especially where hygiene standards are inadequate and bird density is high [91].

## 3. Airborne

Airborne transmission refers to the spread of pathogens through the air, typically via respiratory droplets, dust particles, or aerosolized secretions [92]. This route is especially critical in densely populated poultry environments, where inadequate ventilation and overcrowding significantly increase the risk of disease spread. Airborne transmission allows pathogens to disperse rapidly and over considerable distances, posing a major threat to flock health and public safety. Proper ventilation, rigorous biosecurity protocols, and environmental control measures are essential for minimizing airborne transmission [93,94,95]. Poor air circulation and high stocking densities exacerbate this risk, highlighting the importance of maintaining effective airflow, sanitation, and hygiene practices in poultry facilities [27,96,97,98,99]. Environmental factors, such as wind, temperature, and humidity, influence the survival time and dispersal range of airborne pathogens. Many viral and bacterial agents affecting poultry can spread through the air, underscoring the need for strict control strategies [98]. A summary of the relevant data is presented in Table 1.

Dust particles can carry pathogens, such as AI viruses. External factors—including wind, humidity, temperature, and the type of ventilation system—significantly affect both the infectivity and transmission range of the virus [39].AI can spread through respiratory droplets and aerosolized particles originating from contaminated feces and environmental exposure to polluted areas. These routes are particularly relevant during periods of increased wild bird migration [39,100]. Modeling studies have suggested that AI viruses may travel hundreds of miles under favorable environmental conditions [101].

NDV shares transmission mechanisms with AI. Although it survives in the air for a short time, it can persist long enough in poorly ventilated environments to facilitate infection [47].

Other notable diseases with airborne transmission include ILT and MD [102]. ILT is highly contagious and can survive in the air for several hours and can travel several hundred meters under optimal conditions [27].

MDV demonstrates remarkable environmental persistence, especially under dry conditions. It can remain infectious for up to three weeks at 37.5 °C, eight months at room temperature (22–25 °C), and over three years at 4 °C when associated with feather dust and dander. However, its survival is significantly reduced in humid environments [102].

*E. coli* also contributes to airborne transmission challenges. While *E. coli* has a short airborne survival time (approximately six minutes), it can survive for up to 9.6 h once deposited on surfaces [103]. One study found that *E. coli* could travel up to 800 m downwind in outdoor environments, with measurable concentrations at various distances that gradually decreased with distance. Within poultry houses, *E. coli* concentrations were significantly higher than those recorded outside [104].

*P. multocida*, the causative agent of fowl cholera, also exhibits some airborne persistence. Some *P. multocida* bacteria remain viable after 45 min of exposure [47].

*S. aureus* can survive in aerosolized particles and dust. Once settled in a dry environment, it can remain viable for several months, posing an ongoing risk of infection to both poultry and humans [105,106].

aMPV is primarily transmitted through airborne pathways via aerosolized respiratory secretions. The virus primarily targets the ciliated epithelial cells of the upper respiratory tract, which facilitates efficient airborne spread. Despite being highly contagious, aMPV is relatively short-lived outside the host and is quickly inactivated in the environment [107].

A study demonstrated that fowl adenovirus serotype 4 (FAdV-4) can also be transmitted through aerosols. In controlled experiments, viral aerosols were detected in the isolators two days post-infection, peaking on day four. Healthy birds exposed to these aerosols became infected by day eight, confirming that FAdV-4 can spread efficiently through the air [59].

MS can spread through the air, posing a significant risk to poultry farms. The bacterium primarily infects poultry via the respiratory tract and spreads through both direct and indirect contact. Research has demonstrated that MS can survive for up to 9 days on synthetic hair, indicating its ability to persist in airborne particles [108].

**Table 1:** Summary of economically important airborne pathogens.

Airborne	Distance	Survival Time	Transmission Route	Additional Data
Avian Influenza (AI)	Potentially hundreds of miles [101]	4 °C: more than 900 days 20 °C: 226–293 days 30 °C: 51–58 days	Carried by respiratory droplets and dust particles; risk increases during bird migration [39,100]	
Newcastle Disease (ND)	Limited in poor ventilation [109]	−20 °C: At least 6 months in the bone marrow and muscle of slaughtered chickens. 4 °C: survives over a year 20–25 °C: 30–90 days	No data	Poorly ventilated environments increase transmission risk [109]
Infectious Laryngotracheitis (ILT)	hundreds of meters under optimal conditions [27]	several months in dry dust	No data	
*Escherichia coli*	800 m outdoors [104]	6 min airborne, 9.6 h on surfaces [103]	No data	Higher concentrations indoors; survival depends on environmental conditions [104]
*Pasteurella* *multocida*	No data	45 min [47]	No data	
*Staphylococcus* *aureus*	No data	No data	Remains viable in settled dust for months [105,106]	
Marek’s Disease (MD)	No data	20–25 °C: MDV remains infectious for at least several months. 4 °C: The virus can survive and remain infectious for years [110]	The virus spreads through feather dust and dander [102]	Survival is reduced in humid environments [102]
Avian metapneumovirus (aMPV)	No data	Weeks at 4 °C, 4 weeks at 20 °C, 2 days at 37 °C, and 6 h at 50 °C [47]	Only contact spread has been confirmed	
*Mycoplasma synoviae (MS)*	several km	9 days on synthetic hair, indicating its ability to persist in airborne particles [108]	Lateral transmission occurs readily by direct contact, via the respiratory tract	Infection may also occur as a result of environmental contamina- tion or fomites [47]
*Mycoplasma gallisepticum (MG)*		4 days on feathers, 6 h in the air 3 days on human hair MG isolates can survive inside the human nose for up to 1 day	Airborne transmission via respiratory and conjunctival routes	

## 4. Fomite

Effective sanitation measures, including the regular cleaning and disinfection of equipment and surfaces, are critical for controlling fomite-based transmission routes [19]. Farms lacking adequate cleaning, disinfection programs, and biosecurity protocols are particularly vulnerable to this form of transmission, which involves the spread of pathogens via contaminated surfaces, equipment, litter, and materials. This route is especially concerning because it allows pathogens to persist in the environment for extended periods, facilitating indirect transmission between flocks [111,112]. A summary of the data is presented in Table 2.

Contaminated feather debris and feces can act as passive carriers of AI virus, promoting their spread throughout poultry facilities and introducing the pathogen into feed, water, and soil [47]. AI has a remarkable capacity for environmental persistence—surviving up to 5 days at 24 °C and up to 8 weeks at 4 °C—making temperature a critical factor in its survival [41].

ND virus can persist in litter for days at room temperature, with increased survival at high humidity and temperatures between 0 and 1.7 °C [40,47].

IBV demonstrates even greater environmental stability, surviving in the feces during colder winter months [47]. The ILT virus is spread through respiratory droplets and can persist for up to three weeks on contaminated surfaces and carcasses [27,47].

Similarly, IBD, or Gumboro disease, spreads via fecal shedding and exhibits exceptional environmental resilience. The IBD virus resists various disinfectants, tolerates a wide pH range, and is highly heat-stable. It can survive for up to 16 days in fecal matter, and poultry houses that previously housed infected flocks can remain infectious for several weeks [113].

MD virus is shed through feather dust and dander. The virus exists in an enveloped form within the feather follicle epithelium, allowing it to persist in the environment. Studies have detected viable viruses on dried feathers stored at room temperature for up to 8 months [102,114].

*M. gallisepticum* and *MS* are bacterial pathogens capable of surviving outside the host. *M. gallisepticum* can survive 2 to 4 days on feathers, while strain PG31 has been observed to remain viable for 4 days in feed and 2 days in tap water. *M. synoviae* can persist for 2 to 3 days on feathers [115].

*Salmonella* spp. pose a high environmental risk due to their prolonged survival, lasting up to 291 days in fine manure dust particles [116].

*E. coli* also persists in the environment, often spreading through feces, contaminated water, and poor-quality or improperly stored feed ingredients [62]. This bacterium can survive for over 28 days on stainless steel surfaces under both refrigerated and room temperature conditions [103].

*P. multocida*, the causative agent of fowl cholera, can be shed through feces and contaminate surfaces, water, and feed. This bacterium is known to persist in environmental reservoirs [47].

*C. jejuni* spreads via fecal shedding and can survive up to 4 days in feces under favorable conditions, including low temperatures (10–20 °C) and microaerophilic environments [117,118].

*E. rhusiopathiae* is another environmentally persistent pathogen. It thrives under slightly anaerobic conditions at around pH 7 and can survive for more than a month in the soil [47,79].

Spore-forming bacteria like *C. perfringens* present additional challenges. Wet litter and high bird density promote the risk of infection, particularly in connection with litter quality and peaking behavior [119]. Spores of *C. perfringens* have been shown to survive on stainless steel surfaces for up to 48 h, posing a risk of cross-contamination and potential foodborne transmission [120].

Parasitic infections also contribute to the environmental transmission of pathogens. *H. meleagridis*, the causative agent of blackhead disease in turkeys, is transmitted through the ingestion of infected *H. gallinarum* eggs, which act as biological vectors. These eggs can survive in litter and soil under moist and warm conditions, significantly increasing the risk of transmission [121,122]. In contrast, without its host, *H. meleagridis* survives for only up to 9 h in moist media, feces, or water [123].

*Coccidiosis*, caused by *Eimeria* spp., also spreads via the fecal-oral route. Infected birds shed oocysts that contaminate the litter, soil, water, and feed [124]. These oocysts are highly resilient and can withstand environmental stressors, rapidly sporulating in warm and humid conditions, and maintaining infectivity for extended periods [125].

Mites can spread through contaminated equipment, people, rodents, and wild birds. They can also survive in empty poultry houses for up to 4 weeks, particularly at cooler temperatures, allowing them to persist between flocks [126].

APV RNA has been shown to persist for up to 90 days in autoclaved litter at −12 °C and 8 °C. Viable virus was recoverable for up to 60 days at −12 °C. In non-autoclaved litter, viral RNA remained detectable for 60 days, although viable virus was recoverable for only 14 days, indicating that APV can persist under cold environmental conditions [127].

AEV primarily spreads through the fecal-oral route and can survive in the environment for weeks. Contaminated feed, water, litter, and fomites contribute to its transmission [47].

CAV spreads horizontally through the fecal-oral route and possibly through the respiratory tract. It is also shed from the infected feather follicle epithelium. Contaminated litter serves as a significant source of infection, and the virus has been detected in various organs and rectal contents for up to 35 days post-infection [55].

ARV has also demonstrated environmental persistence. An experimental study found that viable ARV could survive on eggshells for at least 10 days in the presence of organic material. It lasted over 10 days on feathers, wood shavings, and chicken feed, but only 2 days on wood, and 4 days on paper and cotton [128].

**Table 2:** Summary of economically important fomite-transmissible pathogens.

Fomite	Survival Time	Additional Data
Avian encephalomyelitis virus (AEV)	several weeks [47]	No data
Avian Influenza (AI)	5 days at 24 °C; 8 weeks at 4 °C [41]	Feces contamination spreads AI; survival depends on temperature [41]
Avian pneumovirus (APV)	APV can survive in turkey litter for up to 60 days at −12 °C [127]	No data
Avian reovirus (ARV)	In experimental study, 10 days on eggshells when organic material was present >over 10 days—on feathers, wood shavings, and chicken feed only 2 days on wood and 4 days on paper and cotton [128]	No data
Chicken anemia virus (CAV)	Various organs and rectal contents for up to 35 days post-infection [55]	Via fecal-oral route, infected feather follicle epithelium, and possibly via the respiratory tract [55]
Infectious Bronchitis Virus (IBV)	weeks in feces during winter [47]	High resilience in feces
Gumboro Disease (IBD)	16 days in feces; >122 days in poultry houses [129]	Resistant to disinfectants; tolerates wide pH range
Marek’s Disease (MD)	8 months on feathers [110,114]	The virus survives well in environmental reservoirs [110,114]
Newcastle Disease (ND)	weeks in litter [40,47]	Longer survival at low temperatures [40]
*Clostridium perfringens*	Up to 48 h on surfaces [120]	Forms spores that persist on surfaces [119]
*Escherichia coli*	>28 days on stainless steel [103]	No data
*Mycoplasma* *gallisepticum*	2–4 days on feathers, feed, or water [115]	Transmitted via contaminated equipment or water [115]
*Mycoplasma* *gallisepticum* PG31	4 days in feed and 2 days in tap water with 1% culture suspension [115]	No data
*Mycoplasma synoviae*	2 to 3 days on feathers [115]	No data
*Pasteurella multocida*	Variable; persistent in organic material [47]	No data
*Salmonella enterica*	Up to 291 days in manure dust [116]	No data
*Eimeria*	Coccidiosis oocysts survive a few hours [125]	No data

## 5. Waterborne

Ensuring high water quality is fundamental to preventing the waterborne transmission of pathogens in poultry production. This transmission route is especially critical in systems that use shared drinking lines or where water hygiene is poorly maintained. Waterborne transmission refers to the spread of infectious agents through contaminated water sources [19]. Such contamination often results from feces, secretions, or environmental exposure, particularly in intensive farming systems [29]. A summary of the data is presented in Table 3.

AI viruses can survive in water for up to 21 days at 20 °C. Environmental factors, especially temperature and pH, play vital roles in determining the persistence of these compounds in aquatic environments [130].

NDV exhibits similar environmental sensitivity. It can survive in water for 11 to 19 days, depending on conditions such as temperature, disinfectant concentration, presence of organic matter, and pH levels. NDV is more stable at slightly alkaline or neutral pH [131].

IBD also demonstrates remarkable survival in water. It can survive for up to 52 days in water [132].

*M. gallisepticum* has a shorter persistence in water, surviving for 1 to 5 days in pure water and up to 10 days in nutrient-enriched water [133].

The ILT virus is transmitted through respiratory secretions or droplets from infected birds and can contaminate water sources. Notably, it can persist within biofilms that form in drinking water systems, making eradication particularly challenging [47].

Bacterial pathogens, such as *S. enteritidis* and *E. coli,* also thrive in poultry drinking water systems under favorable conditions. *Salmonella* can persist due to its ability to form biofilms inside water lines. Environmental conditions, such as elevated water temperatures (27–30 °C), low water flow, and the presence of nutrient-rich additives, support biofilm development [134,135]. *E. coli* is commonly shed in the feces of infected birds and can enter water systems via fecal contamination, facilitating fecal-oral transmission [136,137]. *P. multocida* exhibits similar environmental persistence, surviving up to 14 days in distilled water at 4 °C and for approximately 49 days at 37 °C [138].

*C. jejuni* is highly sensitive to temperature, showing greater persistence at lower temperatures (10–16 °C), which enhances its survival in water systems during cooler conditions [139].

E. rhusiopathiae can survive in water contaminated with saliva, nasal secretions, or feces. Its ability to persist in diverse environments, including soil and water, illustrates its adaptability and potential for indirect transmission [140].

*C. perfringens* is extremely resilient, capable of surviving for months in water. Its spores contribute to long-term contamination risks in poultry environments [141].

ARV can also persist in contaminated drinking water systems. Studies have shown that ARV remain viable for up to 10 weeks in drinking water, with only minimal reduction in infectivity, supporting continued transmission through the fecal-oral route [128].

**Table 3:** Summary of economically important water transmissible pathogens.

Waterborne	Survival Time	Additional Data
Avian Influenza (AI)	21 days at 20 °C	Persistence influenced by pH and Temperature [142]
Avian reovirus (ARV)	10 Weeks [128]	No data
Infectious Bronchitis Virus (IBV)	52 days [132]	No data
Newcastle Disease (ND)	several days [47]	Stable at neutral/alkaline pH [131]
*Clostridium perfringens*	Months [141]	No data
*Escherichia coli*	Weeks [62,137]	No data
*Mycoplasma* *gallisepticum*	1–10 days [143]	Persistence is favored in nutrient-rich environments
*Pasteurella* *multocida*	14 days at 4 °C; 49 days at 37 °C [138]	Survival increases with increasing temperature
*Salmonella Enteritidis*	No data	Biofilm formation in water lines allows extended survival, especially in warm conditions (27–30 °C) [135,144]

## 6. Vector-Borne

**Figure 2:**
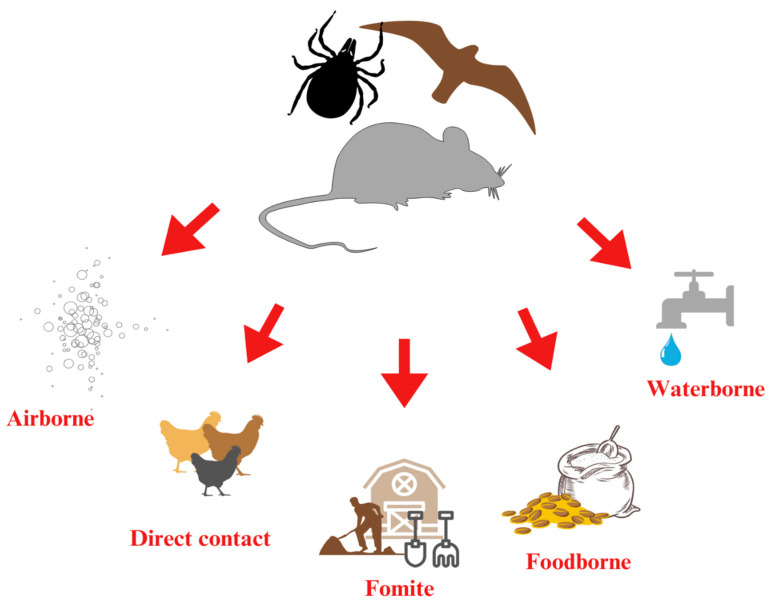
The role of vectors as primary agents in pathogen transmission pathways.

Effective biosecurity measures—including exclusion strategies, habitat management, and targeted control programs—are essential to minimize exposure and prevent the spread of vector-borne diseases in poultry systems [19,23]. Vector-borne transmission refers to the spread of pathogens through intermediate hosts, such as insects, rodents, and wild birds [19]. This transmission route is particularly significant because it enables pathogens to travel across distances and, once introduced, allows further spread via various other transmission pathways [145] (Figure 2). Rodents are major contributors to disease transmission in poultry farms. As both reservoirs and vectors, they can reintroduce pathogens into facilities even after cleaning and disinfection, thereby maintaining a persistent source of infection [146]. A summary of these data is presented in Table 4.

Darkling beetles (*Alphitobius diaperinus*) and mealworms are recognized vectors for several important poultry diseases, including ILT, *S. Typhimurium*, *E. coli*, MD, IBD, pasteurellosis, and coccidiosis [147,148]. Darkling beetles can harbor the ILT virus for up to 42 days [149]. *S. Typhimurium* has been detected in the feces of adult beetles for up to 28 days, and even dead beetles can carry the bacteria for up to 45 days [150]. *E. coli* may persist in or on beetles for up to 12 days and be shed in feces for 6 days in larvae and 10 days in adults [151]. Adult beetles can carry IBDV for at least 14 days post-ingestion [152]. *D. gallinae* poses a unique vector threat, particularly in the transmission of *Salmonella* spp. These mites can harbor Salmonella for prolonged periods of time [153,154,155]. Rodents—especially house mice and rats—can asymptomatically carry pathogens such as *Salmonella* and *C. jejuni* in their intestinal tracts. This allows them to contaminate the environment through feces, urine, and contact with feed, water, and surfaces, despite showing no clinical signs [146].

*D. gallinae* also acts as a vector for several avian pathogens, including *S. enterica*, *E. rhusiopathiae*, *E. coli*, and AI viruses [154,156]. It transmits pathogens through the ingestion of infected blood, mechanical transmission via surface contact, and potentially through vertical transmission [155]. *S. enterica* has been shown to persist in mites for up to four months, while *P. multocida* may survive for 64 to 300 days within these mites [155,156]. Stored mites can remain viable for up to 84 days at 5 °C [157]. Their remarkable survival ability, coupled with widespread resistance to acaricides, complicates control efforts and has led, in some cases, to the use of unauthorized chemical treatments [155]. Wild birds are important vectors of AI, NDV, *H. meleagridis*, and *O. sylviarum*, particularly during migration. Infected birds may contaminate water sources, soil, and surfaces with feces, thereby introducing pathogens into poultry environments [39,121,158].

Migratory birds also contribute significantly to the spread of aMPV. Outbreaks often coincide with migration periods, and the presence of antibodies in species such as geese, sparrows, gulls, parakeets, and various waterfowl suggests ongoing viral circulation among wild birds [107].

FWPV is primarily transmitted via mechanical vectors, with mosquitoes serving as the main carriers. After feeding on infected birds, mosquitoes retain the virus on their mouthparts and transmit it to other birds during subsequent bites. Transmission risk is heightened in warm, humid environments that support high mosquito densities, often following seasonal patterns. Other biting insects, including mites, may also contribute to virus spread. Bird and insect population densities both influence transmission dynamics, making mosquito control—such as eliminating standing water—an essential part of biosecurity strategies [159,160].

In addition to standard biosecurity strategies, integrated vector management approaches are crucial for effective control. These include the regular monitoring of vector populations, targeted application of environmentally safe insecticides or acaricides, and the use of biological control agents, such as predatory insects or entomopathogenic fungi. Structural modifications—such as sealing entry points, installing screens, and improving ventilation—can also reduce vector access to poultry houses. Moreover, environmental management practices like removing organic waste, maintaining dry litter, and minimizing light sources at night can disrupt vector breeding cycles and behaviors. Combining these interventions with ongoing education and training for farm personnel can significantly enhance long-term vector control and reduce the risk of disease transmission [161].

**Table 4:** Summary of economically important vector transmissible pathogens.

Vector-Borne	Survival Time	Additional Data
Avian Influenza (AI)	No data	Transmitted via wild birds and rodents [39]
Avian metapneumovirus (aMPV)	No data	Migratory birds significantly contribute to the spread [127]
Fowlpox virus (FWPV)	No data	Transmitted via biting insects such as mosquitoes and mites [160]
Infectious Laryngotracheitis (ILT)	Darkling beetles harboring ILT for up to 42 days [47]	No Data
*Escherichia coli*	6–12 days in vectors [152]	Spread via beetles and rodents [162]
*Pasteurella multocida*	64–300 days in mites [155]	Mites serve as long-term reservoirs of the pathogen [155]
*Salmonella enterica*	Up to 4 months in mites (*Dermanyssus gallinae*) [156]	Mites act as vectors; wild birds contribute to contamination through feces and physical contact [156]
*Salmonella* *Typhimurium*	28 days in beetles’ feces; 45 days in non-living Beetles [163]	Transmitted by darkling beetles (*Alphitobius diaperinus*) [163]

## 7. Vertical Transmission

**Figure 3:**
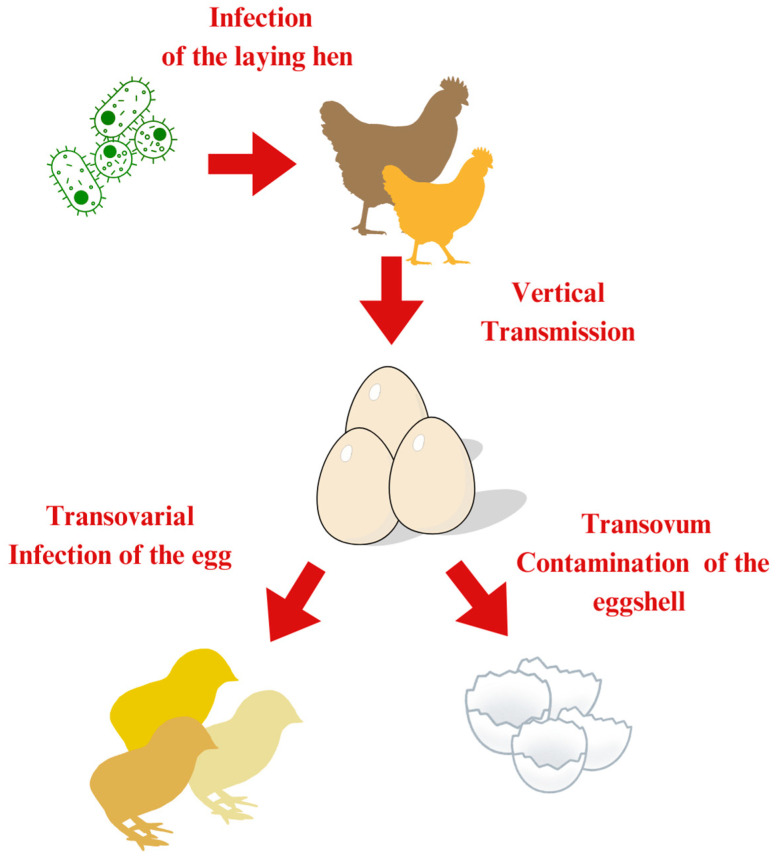
Pathways of vertical transmission illustrating the direct transfer of pathogens to chicks and indirect transmission via contaminated eggshells [166].

Vertical transmission refers to the direct transfer of pathogens from a parent organism to its offspring, typically occurring during egg formation or as the egg passes through the reproductive tract (Figure 3) [164,165]. This transmission route is especially significant in poultry because it ensures the survival and spread of pathogens across generations [62,165]. A summary of the data is presented in Table 5.

For instance, *M. gallisepticum* and *S. Enteritidis* can be transmitted transovarially by infected hens, leading to infected chicks that hatch and continue to spread the disease [32,143,167]. Another *Salmonella* species, *S. infantis*, can also be transmitted vertically. Infections can occur both internally and externally. Through vertical transmission, the bacteria infect the developing ovum transovarially, contaminating the albumen or vitelline membrane before the shell forms. Externally, *S. infantis* can be introduced during or after egg-laying via an infected oviduct, contact with contaminated feces in the cloaca, or exposure to contaminated surfaces in the hen house [168]. This contamination allows pathogens to penetrate the eggshell, leading to embryonic infections and hatchery contamination, which facilitates early chick infections like omphalitis and salmonellosis [19]. *E. coli* follows a similar pattern of vertical transmission, with bacteria passed from hens to chicks during egg formation or as the egg passes through the cloaca [62].

In contrast, vertical transmission of *C. jejuni* is less common, although some studies suggest that it may be a potential route of transmission [169,170].

AEV can be transmitted vertically from infected hens to their offspring via transovarian transmission, resulting in neurological symptoms in hatchlings. Chicks infected in this manner typically show prominent neurological signs, such as tremors, ataxia, and depression, shortly after hatching [54].

Roosters infected with CAV can shed the virus in their semen and transmit it to hens during mating. Hens without immunity become infected with CAV and pass it to their eggs until they develop sufficient antibodies to stop the transmission. Chicks hatched from these eggs are already infected and can spread the virus to other hatchmates who lack immunity [171].

EDS 76 can be transmitted vertically when the primary breeding stock becomes infected, allowing the virus to pass through the egg to the offspring. In its classic form, EDS 76 leads to seemingly healthy birds that later develop egg production issues, including the production of thin-shelled, soft-shelled, or shell-less eggs. Once introduced into a flock through vertical transmission, the virus can spread horizontally through contaminated eggs, equipment, droppings, and personnel, leading to endemic infections in commercial layer farms [61].

FAdV can also spread vertically from parent birds to their offspring. Vertical transmission is an important characteristic of this virus and contributes to its persistence and spread within poultry populations. Studies have confirmed that FAdV can be transmitted from infected breeders to progeny, making it a key factor in the challenges of disease control [60,172].

*M. synoviae* can be vertically transmitted through transovarian transmission. However, the infection rate in breeder hens is relatively low, and some chicks may hatch free of infection despite exposure. Once vertically transmitted, infected chicks can serve as a source of infection for other birds in the flock, further contributing to the spread of MS in poultry facilities. Although vertical transmission plays a role in the persistence of the disease, horizontal transmission via the respiratory tract remains the primary mode of spread within flocks [167,173].

**Table 5:** Summary of economically important vertically transmissible pathogens.

Vertical Transmission	Type of Vertical Transmission
Avian encephalomyelitis virus (AEV)	Transovarian transmission [54]
*Avian influenza*	There is some evidence of vertical transmission; however, infected eggs are unlikely to hatch successfully [39]
Avian reovirus (ARV)	Transovarial and transovum transmission [174]
Chicken anemia virus (CAV)	Transovarian transmission [55]
Egg drop syndrome 76 (EDS 76)	Transovarian transmission [61]
Fowl adenovirus (FAdV)	Transovarian transmission [175]
*Campylobacter jejuni*	Potential but rare vertical transmission; more often transmitted via fecal-oral routes [169,170]
*Escherichia coli*	Transovarial and transovum transmission [62]
*Mycoplasma gallisepticum*	Transovarial transmission [167]
*Mycoplasma synoviae*	Transovarian transmission (infection rate is low) [65,167]
*Salmonella enteritidis*	Transovarial transmission [32,143]
*Salmonella infantis*	Transovarial and transovum transmission [168]

## 8. On-Farm Biosecurity and Disinfectants

Proper biosecurity is essential in large-scale poultry farms, both at the breeding and commercial levels. The implementation of the following biosecurity systems is a prerequisite for the efficient and safe operation of large-scale poultry farms. A robust three-dimensional biosecurity system for poultry farms encompasses external, internal, and procedural-operational biosecurity, each of which plays a critical role in preventing the introduction and dissemination of pathogens both within and beyond poultry facilities [176,177,178].

External biosecurity (perimeter level) includes measures to prevent the entry of infectious agents from external sources, such as personnel, vehicles, equipment, feed, and wild animals, into the farm environment [17,179]. This includes the regulation of visitor and staff movements, controlled farm access with hygiene barriers (e.g., Danish entry systems) [180]. The use of personal protective equipment (PPE), decontamination protocols, traffic control, and vehicle and equipment disinfection protocols [181,182], restrictions on the import of animals and animal products, procurement of poultry (live animals), and feed from certified pathogen-free sources [176,178] disinfection of incoming goods, and the prevention of contamination via feed, water, pests, or wild birds, and pest and wild bird control to limit contact with potential reservoirs of avian pathogens [183].

Internal biosecurity measures (zonal level) aim to minimize the spread of infectious agents within an animal farm. Key practices include cleaning and disinfecting facilities, especially between production cycles, and water and litter management to reduce microbial load; barn-specific personal protective equipment (PPE), zoning, and compartmentalization to isolate different age groups or health statuses, managing stocking densities, following all-in-all-out procedures to reduce cross-contamination between flocks, and separating susceptible or diseased individuals [19,179,184,185,186].

In the context of procedural and operational biosecurity (personnel measures level), staff behavior and adherence to farm protocols play crucial roles in pathogen control. Training programs on hygiene practices and disease recognition are crucial for disease prevention and the proper implementation of biosecurity measures on animal farms. In addition, monitoring and recording systems to trace health and production parameters, and the above-mentioned use of personal protective equipment and strict clothing/footwear change protocols between different zones are a part of the ideal operation of the biosecurity system of large-scale poultry farms [19,47].

In addition, the integration of surveillance systems, including pathogen monitoring and risk-based sampling, contributes to the early detection and containment of infectious diseases. Modern approaches also emphasize the use of digital technologies, such as biosensors, artificial intelligence-driven disease forecasting, and remote monitoring, to strengthen global biosafety infrastructure [47,176,177,178].

To support the global applicability of such systems, organizations such as the World Organisation for Animal Health (WOAH, formerly OIE) and the FAO have published guidelines and frameworks for harmonized biosecurity standards adaptable across different geographic and production contexts [176,178].

Disinfectants play a vital role in biosecurity by inactivating pathogens on surfaces, equipment, and within the environment [187]. Selecting the appropriate disinfectant, along with the proper concentration, contact time, and application method, is critical for addressing a wide range of threats, including viral and bacterial pathogens [19].

For ILT, a combination of chemical and physical disinfection methods is often required. Common disinfectants, such as 3% cresol, 5% phenol, and 1% sodium hydroxide, can inactivate the ILT virus within one minute. However, the presence of organic materials, such as litter or respiratory secretions, can reduce their effectiveness. The ILT virus is sensitive to heat and can be effectively inactivated by heating at 55 °C for 15 min or composting at 38 °C for 24 h [27].

IBDV exhibits high resilience to environmental stressors. Disinfectants such as Virkon, surface decontamination foam (SDF), and bleach have shown effectiveness, although to varying degrees. Virkon demonstrated the highest efficacy, achieving significant inactivation at −20 °C within 2 h. SDF required 24 h to reach a similar effectiveness, while bleach was less effective, requiring 24 h at −25 °C. Virkon has proven particularly efficient in the presence of organic matter [188].

MDV can be inactivated using several disinfectants, including chlorine, quaternary ammonium compounds, organic iodine, cresylic acid, synthetic phenol, and sodium hydroxide. These agents are capable of destroying the virus on contaminated feathers within 10 min [102].

In contrast, *M. gallisepticum* exhibits moderate resistance to disinfection and requires specific compounds for effective inactivation. Quaternary ammonium compounds (QAC), phenolics, and chlorine-based products have proven to be among the most effective [189].

Effective disinfection against *Salmonella* in poultry facilities involves a multi-step approach. High-temperature cleaning with surfactants at 65 °C, followed by rinsing with water at 80 °C, significantly reduces bacterial loads. Chemical disinfectants, such as chlorine dioxide and dolomitic lime, are particularly effective. Dolomitic lime contributes to the creation of an alkaline environment that disrupts biofilms and bacterial survival [190].

*P. multocida* is effectively inactivated by the combination of glutaraldehyde and QAC, resulting in a significant reduction of *P. multocida* after 15 min of exposure [191]. *C. jejuni* is susceptible to benzalkonium chloride, P-128, and ammonium chloride-based disinfectants [192].

*E. rhusiopathiae* is effectively inactivated by sodium hypochlorite and sodium hydroxide (NaOH). However, it shows resistance to alcohols, aldehydes, oxidizing agents, and phenols. The removal of organic material prior to disinfection is essential to ensure optimal effectiveness [193].

*C. perfringens* requires a combination of mechanical and chemical methods. Effective approaches include pressure washing, application of 5% sodium hypochlorite or quaternary ammonium compounds, and maintaining prolonged drying periods of up to 48 h [194].

For highly contagious viruses, such as AIV and NDV, oxidizing disinfectants like Virkon and Accel are recommended. Their efficacy is enhanced when combined with agents such as calcium chloride, methanol, or propylene glycol. For example, Virkon with 20% calcium chloride inactivates the virus within 5 min. Similarly, Accel with 20% calcium chloride or methanol achieves full inactivation within the same timeframe. These combinations provide fast and reliable virus elimination under various environmental conditions [188].

For IBV, disinfectants containing potassium peroxymonosulfate alone or in combination with sodium dodecylbenzenesulfonate, sulfamic acid, and inorganic buffers are highly effective. Potassium peroxymonosulfate is particularly efficient, acting within 30 min at a 1:200 dilution and even more rapidly at 1:100, making it the preferred choice [195].

aMPV can be effectively inactivated by quaternary ammonium compounds, ethanol, iodophors, phenol derivatives, and sodium hypochlorite, all of which significantly reduce viral viability [51].

Fowlpox virus, including both wild-type and vaccine strains, is rapidly inactivated—within one minute—when exposed to 70% ethanol, 50% isopropyl alcohol, 0.5% sodium hypochlorite, 30% formaldehyde, 10% benzalkonium chloride, a mixture of 6.67% cetyltrimethylammonium chloride and 3.33% benzalkonium chloride, or a combination of 1.75% iodine with 10% polyethylene glycol nonylphenyl ether [196].

AEV is highly sensitive to disinfection with 10% isopropyl alcohol and 2% formalin, both of which have been identified as highly effective [54].

ARVs are best inactivated using oxidizing agents and quaternary ammonium compounds combined with aldehydes, achieving virus elimination within 2 to 5 min [197]. A summary of these data is presented in Table 6.

## 9. Role of Social Media in Countering Misinformation

In response to the increasing threat posed by misinformation and disinformation in the field of animal health and biosecurity, the integration of modern communication channels—particularly social media platforms—has become not only advantageous but essential. As the World Organisation for Animal Health (WOAH) and INTERPOL emphasize, misinformation, if left unchallenged, can significantly undermine disease control measures, erode public trust, and jeopardize animal and public health during emergencies.

Social media platforms such as X (formerly Twitter), Facebook, Instagram, and LinkedIn offer veterinary researchers and institutions an unprecedented reach to disseminate science-based, accurate information beyond traditional academic circles. Visual content formats—infographics, short educational videos, and graphical summaries of research findings—can facilitate the rapid and accessible transmission of key messages to diverse audiences, including farmers, policymakers, and the general public [199,200].

In the digital information ecosystem, which is characterized by echo chambers and filter bubbles, proactive communication by trusted scientific actors is vital. Studies have shown that misinformation often spreads faster than accurate content, not only due to malicious actors but also because human users tend to share content that is emotionally engaging or simplistic. Consequently, clear, relatable, and visually engaging communication by veterinary experts can pre-empt the viral spread of falsehoods, a strategy referred to as “pre-bunking”.

Moreover, transparency in communicating scientific uncertainty, contextualizing research findings, and consistently engaging digital audiences fosters credibility. As highlighted in [201], building “cognitive resilience” through awareness-raising, cross-sector collaboration, and training in digital risk communication enhances institutional preparedness and public responsiveness in animal health emergencies.

Social media also enables “social listening”—the monitoring of public sentiment and emerging narratives—which is now recognized as a core component of epidemic intelligence systems. This capability supports timely responses to evolving misinformation and can guide targeted educational interventions [202,203].

## 10. Discussion

The complexity and diversity of pathogen transmission routes in poultry production highlight the critical need for comprehensive and integrated biosecurity measures [204]. This review has emphasized the major transmission pathways, including direct, indirect, airborne, waterborne, vector-borne, and vertical routes, and their implications for flock health, public safety, and the economic sustainability of the poultry industry.

Direct transmission, particularly prevalent in high stocking density systems, facilitates the rapid spread of pathogens such as AIV and NDV [205]. These intensive systems increase the risk due to limited space per bird and environmental stressors that compromise the immune function [206,207]. Mitigating these risks requires enhanced ventilation and adherence to optimal stocking densities [144,208]. and the implementation of stress-reduction strategies, such as nutritional support and reduced overcrowding [207,209].

Indirect transmission poses a substantial challenge due to the environmental persistence of pathogens like IBDV and *E. coli*. These pathogens can survive in litter, feed, and water, underscoring the need for routine cleaning, proper feed storage, and water quality management [19,23]. Effective biosecurity protocols—including equipment and vehicle disinfection, regular waste removal, and the use of appropriate disinfectants—can significantly reduce indirect transmission risks [204].

Airborne transmission, driven by respiratory droplets and dust particles, remains a major concern in densely populated poultry environments [101]. Mitigation strategies include the use of advanced air filtration systems, humidity control, and improved ventilation to reduce the spread of pathogens. Additionally, maintaining a clean housing environment to minimize dust generation is essential [23].

Waterborne transmission, primarily via shared drinking systems, highlights the importance of a clean water supply. Pathogens such as AI and IBDV can survive for extended periods in water, necessitating routine testing, biofilm control in water lines, and regular disinfection of water systems [29,132].

Vector-borne transmission via insects, rodents, and wild birds is another critical threat [29,162]. Vectors such as darkling beetles, red mites, and rodents can harbor and transmit pathogens like *S. Typhimurium*, *E. coli*, and ILT virus [151,163]. Controlling vectors through structural maintenance, exclusion strategies, and habitat management can greatly reduce the risk of pathogen introduction [19].

Vertical transmission—through infected eggs or transovarial routes—further complicates disease control efforts [62]. Pathogens such as *M. Gallisepticum* and *S. Enteritidis* can be transmitted across generations, maintaining infections within breeding populations [32,143,167]. Effective control strategies include routine monitoring and screening of breeder flocks, along with vaccination programs to reduce the pathogen load in parent stock [19].

These findings underscore the necessity of an integrated biosecurity approach—integrating sanitation, environmental management, vector control, and vaccination—to protect poultry health and enhance the sustainability of production systems [19,23]. Future research should prioritize the development and implementation of innovative technologies, such as artificial intelligence (AI)-driven monitoring and predictive systems to anticipate outbreaks and optimize biosecurity responses. By addressing these multifactorial challenges, the poultry industry can meet the increasing global demand while safeguarding public health, food safety, and food security [210].

The intentional use of social media by poultry health researchers not only strengthens the public impact of academic work but also serves a critical function in countering misinformation, safeguarding biosecurity, and promoting evidence-based decision-making in the poultry sector.

## 11. Conclusions

This article highlights the wide range of transmission routes and environmental resilience of pathogens affecting global poultry production. These include airborne transmission, fecal-oral routes, vector-borne pathways, and indirect contamination through food, water, and fomites (contaminated objects or surfaces). Studies have emphasized the critical importance of strict biosecurity protocols, particularly in intensive farming systems, where high stocking densities, confined spaces, and shared resources create ideal conditions for disease outbreaks. A solid understanding of effective control measures, such as improving ventilation, is essential to reduce the survival and spread of airborne pathogens. The use of air filtration systems may help reduce pathogen transmission and further enhance air quality. Pest control measures, including regular disinfection, facility hygiene, proper manure and waste management, and maintenance of a clean and dry environment, are also vital. Secure physical barriers can prevent the entry of disease-carrying vectors. Additionally, addressing stress and supporting immune function in poultry populations is crucial for reducing the risk of opportunistic pathogens. Future research should focus on developing sustainable practices that balance production efficiency and animal health, ultimately protecting public health, food safety, food security, and the economic stability of the poultry industry. Further interdisciplinary research is needed to explore the long-term effectiveness of integrated disease-management strategies across diverse production systems. This includes evaluating the socio-economic impacts of biosecurity interventions, the role of climate change in pathogen ecology, and the development of innovative surveillance technologies, such as environmental monitoring and genomic tools. Insights gained from such studies should inform national and international policy frameworks aimed at strengthening poultry health infrastructure, enhancing cross-border disease reporting, and incentivizing sustainable farming practices that align with the One Health principles. The pathogens discussed in this study were selected based on their significant economic impact on poultry production, diverse transmission pathways, and potential to cause widespread disease outbreaks. Poultry health and productivity are under constant threat from viral, bacterial, and parasitic pathogens—many of which exhibit high transmission rates, environmental persistence, and resistance to control measures. HPAI and NDV were included due to their devastating effects on poultry populations, zoonotic potential, and their role in triggering global trade restrictions. Other important viral pathogens, such as IBV, MDV, ILT, and aMPV, were included for their ability to spread rapidly through airborne transmission and cause severe respiratory or immunosuppressive diseases. IBD and CIA were selected due to their immunosuppressive effects, which increase susceptibility to secondary infections EDS-76 and FAdV were included due to their impact on egg production and quality. Among bacterial pathogens, *E. coli, S. Enteritidis, S. Infantis*, *S. Typhimurium*, *M. synoviae*, and *M. gallisepticum* were selected for their roles in systemic and respiratory infections, persistence in poultry environments, and zoonotic potential. Other bacterial threats, including *P. multocida*, *C. perfringens*, *C. jejuni*, *Staphylococcus aureus*, and *E. rhusiopathiae*, were included due to their ability to cause high mortality, decreased feed efficiency, reduced growth performance, and poor egg production, all contributing to significant economic losses. The emergence of antibiotic-resistant strains, such as MRSA, further underscores the need for stringent biosecurity and disease prevention measures. Parasitic pathogens such as *Eimeria* spp., *D.s gallinae*, *O. sylviarum*, and *H. meleagridis* were also included due to their direct effects on poultry health and productivity, and their potential to facilitate secondary infections.

## Figures and Tables

**Table 6:** Summary of effective disinfectants against specific pathogens.

Pathogen	Effective Disinfectants
Avian encephalomyelitis virus (AEV)	10% isopropyl alcohol and 2% formalin [54]
Avian influenza virus (AI)	Virkon (oxidizing agent), Accel (hydrogen peroxide-based), Cresol (3%) and Phenol (5%) [188]
Avian metapneumovirus (aMPV)	quaternary ammonia, ethanol, iodophor, phenol derivatives, and sodium hypochlorite [51]
Avian reoviruses (ARV)	Quaternary ammonium compounds combined with aldehydes [197]
Fowlpox virus (FWPV)	70% ethanol, 50% isopropyl alcohol, 0.5% sodium hypochlorite, 30% formaldehyde, 10% benzalkonium chloride, a combination of 6.67% cetyltrimethylammonium chloride and 3.33% benzalkonium chloride, or a mixture of 1.75% iodine and 10% polyethylene glycol nonylphenyl ether [196]
Infectious bronchitis virus (IBV)	Potassium Peroxymonosulfate (e.g., Virkon S), Sodium Dodecylbenzenesulfonate [195]
Infectious bursal disease virus (IBDV)	Virkon, Surface Decontamination Foam (SDF), Bleach (varies in effectiveness) [195]
Infectious laryngotracheitis virus (ILT)	Cresol (3%), Phenol (5%), Sodium Hydroxide (1%) [27]
Marek’s disease virus (MDV)	Chlorine, Quaternary Ammonium Compounds, Sodium Hydroxide [102]
Newcastle disease virus (NDV)	Virkon, Accel, Sodium Hydroxide (1%) [188]
*Campylobacter jejuni*	Benzalkonium Chloride, P-128, Ammonium Chloride-based disinfectants [192]
*Clostridium perfringens*	pressure washing, pressure washing combined with sodium hypochlorite (5%) or quaternary ammonium sprays [194]
*Escherichia coli*	Benzalkonium Chloride, P-128 (ammonium chloride-based)
*Erysipelothrix rhusiopathiae*	Hypochlorite, Sodium Hydroxide, Remove organic matter before application [193]
*Mycoplasma gallispeticum*	Quaternary ammonium compounds, phenolics, and chlorine-based products [198]
*Mycoplasma (synoviae)*	Ethanol and alkaline detergent formulations [198]
*Pasteurella multocida*	combination of glutaraldehyde and quaternary ammonium compounds (QAC) achieved a significant reduction [191]
*Salmonella* spp.	Chlorine Dioxide, Dolomitic Lime, Sodium Hypochlorite [190]
*Staphylococcus aureus*	Quaternary Ammonium Compounds, Sodium Hypochlorite

## Data Availability

The original contributions presented in this study are included in the article. Further inquiries can be directed to the corresponding author(s).

## Abbreviations

The following abbreviations are used in this manuscript:

AEV	Avian encephalomyelitis virus
AI	Avian influenza
aMPV	Avian metapneumovirus
APEC	Avian Pathogenic Escherichia coli
APV	Avian pneumovirus
ART	Avian rhinotracheitis
ARV	Avian Reovirus
CAV	Chicken anemia virus
CIA	Chicken infectious anemia
EDS 76	Egg drop syndrome 76
FAdV	Fowl adenovirus
FAO	Food and Agriculture Organization of the United Nations
FT	Fowl Typhoid
FWPV	Fowlpox virus
HP	Hydrogen Peroxide
HPAI	Highly Pathogenic Avian Influenza
IBD	Infectious Bursal Disease
IBV	Infectious Bronchitis Virus
ILT	Infectious Laryngotracheitis
LPAI	Low Pathogenic Avian Influenza
MD MDV	Marek’s Disease Marek’s Disease Virus
MRSA	Methicillin-resistant Staphylococcus aureus
MS	Mycoplasma synoviae
MT	Metric Tons
NDV	Newcastle disease virus
OECD	Organization for Economic Co-operation and Development
PCV	Packed cell volume
PD QAC	Pullorum disease Quaternary ammonium compounds
SDF	Surface Decontamination Foam
SHS	Swollen head syndrome
TRT	Turkey rhinotracheitis
